# Using the Theory of Planned Behaviour to Describe Male Involvement Intention During Childbirth Among Expecting Couples in a Rural Setting: A Cross-Sectional Study From Rukwa Region, Southern Tanzania

**DOI:** 10.24248/EAHRJ-D-18-00018

**Published:** 2019-07-30

**Authors:** Fabiola V Moshi, Stephen M Kibusi, Flora Fabian

**Affiliations:** a School of Nursing and Public Health, University of Dodoma, Dodoma, Tanzania; b School of Medicine and Dentistry, University of Dodoma, Dodoma, Tanzania

## Abstract

**Background:**

Male involvement during childbirth can increase utilisation of maternal services and reduce maternal and neonatal mortality. An individual's intention towards such male involvement can be understood through the theory of planned behaviour, which postulates that such intention is influenced by 3 domains: 1) attitudes, 2) perceptions of social approval (subjective norms) and 3) feelings about control over the intended behaviour. In sub-Saharan Africa, rates of male involvement in childbirth birth are low, and little is known about the predictors of intention for such involvement among expecting couples in rural Africa. This study aimed to determine the influence of the 3 domains of intention on male involvement intention during childbirth among expecting couples in Rukwa Region, Tanzania.

**Methods:**

We conducted a community-based, cross-sectional study of pregnant women and their partners from June until October 2017. In total, 546 couples (n=1,092 participants) were identified through 3-stage probability sampling. A structured questionnaire based on the theory of planned behaviour was used to elicit information on the 3 domains of intention.

**Results:**

Most pregnant women (71.6%) and their male partners (77.3%) intended to have male involvement during childbirth. Among women, only positive attitude (odds ratio [OR] 0.2, 95% CI, 0.1 to 0.7; *P*=.012) was significantly associated with intention, though in an unexpected direction. In adjusted analysis, men's positive attitude (adjusted odds ratio [AOR] 9.0, 95% CI, 1.9 to 40.9; *P*=.004) and positive subjective norms (AOR 4.4, 95% CI, 1.1 to 18.6; *P*=.041) were significantly associated with an increased likelihood of intention to accompany their partners during childbirth.

**Conclusion:**

More male partners had the intention to accompany their spouses during childbirth compared to their female partners. Male attitudes and subjective norms may be influential in determining male involvement during childbirth in rural African settings.

## INTRODUCTION

An estimated 293,300 maternal deaths occurred in 2013 worldwide.^1^ Most of these deaths occurred in sub-Saharan Africa, where low rates of use of skilled birth attendants are associated with high maternal and neonatal mortality.^2,3^ Birth preparedness, when a mother or couple engages in planning and preparation for childbirth, increases use of a skilled birth attendants^4^ by reducing delays in accessing maternal services, include those related to decision making to seek health care, reaching a health facility and obtaining appropriate care within health facilities.^5^ Male involvement in birth preparedness, can increase utilisation of maternal services and reduce maternal and neonatal mortality.^6-9^ However, in sub-Saharan Africa male involvement in birth preparedness is low: 32.1% in Nigeria;^8^ 42.9% in Uganda;^10^ 18% in Burundi;^9^ and 12% in Tanzania.^11^ Barriers to male involvement in birth preparedness include a lack of collective decision making at the household level, sociocultural beliefs about gender roles and responsibilities, peer influence, and health workers' attitudes towards their involvement.^12-15^

Male involvement in birth preparedness can be understood through the theory of planned behaviour, which links one's beliefs and behaviours. According to this the theory, an individual's intention to be involved during childbirth is influenced by their beliefs (Figure). This includes their attitude towards male involvement, their perception of social pressures regarding male involvement (subjective norms), and the extent to which they feel able to accompany or be accompanied during childbirth (perceived behaviour control). Individuals are more likely to intend to have healthy behaviours if they: 1) have positive attitudes about the behaviours, 2) believe that community norms favour the behaviours, and 3) believe they are able to carry out the behaviours. An individual's intentions will be stronger when they have all 3 of the above elements, compare to when they have only 1.^16^ At present, little is known about the influence of attitudes, subjective norms and perceived behaviour control towards male involvement among expecting couples in Africa. This study aimed to use the theory of planned behaviour^17^ to describe male involvement intention among expecting couples in the rural setting of Rukwa Region, Tanzania.

**FIGURE:**
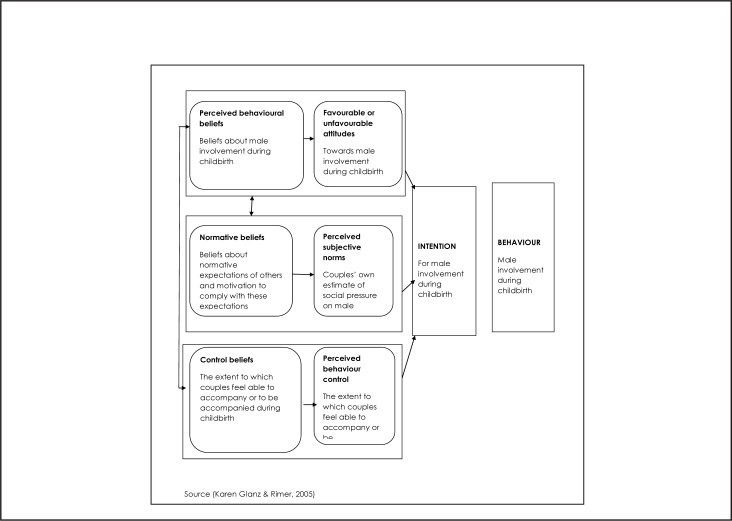
Description of Male Involvement Using Theory of Planned Behaviours

## METHODS

### Study Design and Setting

This study was conducted in Rukwa Region, Tanzania, a country that has among the highest rates of maternal mortality worldwide^18^ with 6 maternal deaths for every 1,000 live births.^19^ With a population of 1,004,539, Rukwa Region has Tanzania's lowest mean age at marriage; men marry at an average age of 23.3 years and women at an average age of 19.9 years.^20^ The region's fertility rate is 7.3 children per woman,^20^ and health facility coverage is low, with just 1 facility for every 2 villages. From June to October 2017, we conducted a cross-sectional community-based survey among expecting couples from 45 villages in Rukwa Region to determine the influence of attitudes, subjective norms, and perceived behaviour control on the intention for male involvement in childbirth.

### Sampling

Two districts (Sumbawanga Rural District and Kalambo District) were purposively selected from the 4 districts of Rukwa Region; these districts were chosen because they have a high proportion of childbirth not attended by a skilled birth attendant. Four-stage cluster sampling was used to obtain study participants. First, 15 villages from Sumbawanga District and 30 from Kalambo District were randomly selected from among all villages in the selected wards. Next, 4 hamlets were randomly selected from each village. Finally, systematic random sampling was used to obtain a maximum of 4 households per hamlet, in which a pregnant woman who was at <25 weeks gestation resided with a male partner. Each hamlet had approximately a total of 30 households and approximately 6 to 8 pregnant women.

### Sample Size

The minimum sample size required to estimate the odds ratio (OR) for the study outcome associated with explanatory factors, was determined using the following formula^21^:

$$n=\frac{{\left[Z\alpha \sqrt{(1+m)\pi o\left(1-\pi o\right)}+Z\beta \sqrt{\pi 1\left(1-\pi 1\right)+m\pi 2\left(1-\pi 2\right)}\right]}^{2}}{{\left(\pi 2-\pi 1\right)}^{2}}$$

where *Zα* is the value 1.96 for a 95% confidence interval (CI), *Zβ* is the value 0.84 for 80% power, π1 is the proportion of controls that have the exposure, π2 is an auxiliary variable equal to OR x π1/(1-π1+OR x π1), πo is (π1+π2)/2, *m* is the number of controls per case, and *n* is the required sample size per case and control group. Here we treated participants with the intention for male involvement as cases and assumed cases would represent 50% of the sample. Our minimum OR of interest was 2, and we assumed that explanatory factors would be prevalent at 15% or more in the control group. The formula yielded a minimum sample size of 420. The study included a total of 546 couples; analyses were conducted separately for men and women.

### Data Collection

For eligibility screening, female partners were asked to undergo a pregnancy test. Women with confirmed pregnancies and their male partners were administered informed consent for study participation; those who consented were enrolled in the study. Trained research assistants conducted in-person interviews with participants using a semi-structured questionnaire about birth preparedness intention, which was developed based on the theory of planned behaviour. The questionnaire assessed sociodemographic characteristics and participants' attitudes, perceived subjective norms, and perceived behaviour control regarding male involvement in childbirth. Questionnaires were administered separately to each half of the participating couples.

### Variable Definitions and Data Analysis

The dependent variable was the intention to be accompanied (pregnant women) or to accompany (male partners) during childbirth, which was coded as ‘Yes’ or ‘No’. The independent variables of interest were participants' attitudes, subjective norms, and perceived behaviour control with regard to male involvement in childbirth, which were assessed using a 5-level Likert-type scale consisting of 12 statement items. These included statements like, “If I participate in setting aside some funds and equipment which will be used in case of an emergency or during delivery I am doing a good thing”; “If I participate in the preparation of transport which will be used in case of emergency or during childbirth I am doing a good thing”; and “If I participate in the identification of skilled attendant I am doing a good thing”. On the Likert scale, 1 indicated “strongly disagree”, 2 indicated “disagree”, 3 indicated “neither agree nor disagree”, 4 indicated “agree”, and 5 indicated “strongly agree”. Factor analysis was done to measure the attitude, perceived subjective norms and perceived behaviour control. The normality test was conducted, and the mean scores were established. The regression score above mean were termed as positive and below mean negative. A positive attitude indicates that the individual expects favourable results from male involvement during childbirth, while a negative attitude indicates that they expect unfavourable results. Positive perceived subjective norms indicate that the participant feels male involvement in childbirth is approved by relatives and friends while negative perceived subjective norms indicate they feel it is disproved. Positive perceived behaviour control indicates that the participant feels they are able to engage in the behaviour, while negative perceived behaviour control indicates the participant feels unable to engage in the behaviour.

Statistical analyses of study data were conducted separately for men and women. Descriptive statistics were generated for sociodemographic and Likert-type scale variables; Pearson's chi-square (Χ^2^) test was used to assess bivariate associations between explanatory variables and intention for male involvement in childbirth. Multivariable logistic models were developed to assess the association between explanatory variables and the outcome variable. Explanatory variables that were significant in the bivariate analysis at *P*>.20 were considered candidates for the multivariable logistic models. The final multivariable logistic models were developed using backwards elimination of explanatory variables with *P* values >.05. Prior to analysis, data were checked for completeness and consistency and entered into a database using statistical package IBM SPSS Statistics for Windows version 23.0 (IBM Corp, Armonk, NY, USA).

### Ethical Considerations

The proposal was approved by the Ethical Review Committee of the University of Dodoma (reference number UDOM/DRP/134 VOL.III/29). A letter of permission was obtained from the Rukwa Regional Administration. Written informed consent was obtained from study participants after explaining the study objectives and procedures. Participants were informed of their right to refuse to participate or withdraw from the study. Each respondent was assigned an identity number, and all collected data were anonymised.

## RESULTS

In total, 546 couples were included in the study. The mean age of pregnant women was 25.5 years, and the mean age of their spouses was 30.7 years. The majority of couples were married (71.4%), monogamous (85.9%), lived on less than 1 dollar per day (70.0%), and received basic obstetric care services from dispensaries (82.8%) (data not shown). Most pregnant women (54.8%) and their spouses (64.7%) had completed only a primary level of education (Tables 1 and 2).

**TABLE 1.** Characteristics of Pregnant Women in Rukwa Region, Tanzania, 2017 (N=546)

		Intend to Have Male Partner Involved With Childbirth		
	Total	Yes	No	
	n	n (%)	n (%)	*P Value*
**Total**	546	391 (71.6)	155 (28.4)	
**Characteristic**				
**Age group, years**				
<20	167	121 (72.5)	46 (27.5)	.058
21-25	156	109 (69.9)	47 (30.1)	
26-30	105	66 (62.9)	39 (37.1)	
31-35	55	43 (78.2)	12 (21.8)	
>35	63	52 (82.5)	11 (17.5)	
**Age at marriage (years)**				
<18	395	279 (70.6)	116 (29.4)	.714
19-24	147	109 (74.1)	38 (25.9)	
>25	4	3 (75)	1 (25.0)	
**Parity**				
0	120	90 (75.0)	30 (25.0)	.014
1-4	320	215 (67.2)	105 (32.8)	
>4	106	86 (81.0)	20 (19.0)	
**Prior pre-term delivery**				
Yes	29	20 (69.0)	9 (31.0)	.745
No	527	371 (70.4)	146 (27.7)	
**Ethnic group**				
Fipa	322	237 (73.6)	85 (26.4)	<.001
Mambwe	120	106 (88.3)	14 (11.7)	
Other	104	48 (46.2)	56 (54.8)	
**Total**	546	391 (71.6)	155 (28.4)	
**Characteristic**				
**Education level**				
None	230	153 (66.5)	77 (33.5)	.072
Primary	299	226 (75.6)	73 (24.4)	
Secondary or higher	17	12 (70.6)	5 (29.4)	
**Individual income per day**				
<1 dollar	399	285 (71.4)	114 (28.6)	.876
>1 dollar	147	106 (72.1)	41 (27.9)	
**Owns radio**				
Yes	253	173 (68.4)	80 (31.6)	.12
No	293	218 (74.4)	75 (25.6)	
**Household owns mobile phone**				
Yes	69	55 (79.7)	14 (20.3)	.110
No	477	336 (70.4)	141 (29.6)	
**Adult female in the family**				
None	318	224 (70.4)	94 (29.6)	.473
>1	228	167 (73.2)	61 (26.8)	
**Total**	546	391 (71.6)	155 (28.4)	
**Characteristic**				
**Covered by health insurance**				
Yes	177	115 (65)	62 (35)	.017
No	369	276 (74.8)	93 (25.2)	
**Nearest health facility**				
Clinic	452	334 (73.9)	118 (26.1)	.010
Dispensary	94	57 (60.6)	37 (39.4)	
**Distance to nearest health facility, km**				
<1	258	177 (68.6)	81 (31.4)	.262
1-5	233	171 (73.4)	62 (26.6)	
>5	55	43 (78.2)	12 (21.8)	
**Attitude towards male involvement in childbirth**				
Negative	7	3 (42.8)	4 (57.2)	.089
Positive	539	388 (72)	151 (28)	
**Subjective norms regarding male involvement in childbirth**				
Negative	36	33 (91.7)	3 (8)	.006
Positive	510	358 (70.2)	152 (29.8)	
**Perceived behaviour control regarding male involvement in childbirth**				
Negative	7	5(71.4)	2 (28.6)	.991
Positive	539	386 (71.6)	153 (28.4)	

**TABLE 2.** Characteristics of Male Partners of Pregnant Women in Rukwa Region, Tanzania, 2017 (N=546)

		Intend to Have Male Partner Involved With Childbirth		
	Total	Yes	No	
	n	n (%)	n (%)	*P Value*
**Total**	546	391 (71.6)	155 (28.4)	
**Characteristic**				
**Age group, years**				
<20	27	24 (88.9)	3 (11.1)	.493
21-25	143	114 (79.7)	29 (20.3)	
26-30	146	112 (76.7)	34 (23.3)	
31-35	87	65 (74.7)	22 (25.3)	
>35	143	107 (74.8)	36 (25.2)	
**Age at marriage, years**				
<18	71	60 (84.5)	11 (15.5)	.065
19-24	353	276 (78.2)	77 (21.8)	
>25	122	86 (70.5)	36 (29.5)	
**Ethnic group**				
Fipa	367	299 (81.5)	68 (18.5)	<.001
Mambwe	118	94 (79.7)	24 (20.3)	
Other	61	29 (47.5)	32 (52.5)	
**Education level**				
None	155	113 (72.9)	42 (27.1)	.142
Primary	353	282 (79.9)	71 (20.1)	
Secondary or higher	38	27 (71.1)	11 (28.9)	
**Income per day**				
<1 dollar	382	290 (75.9)	92 (24.1)	.242
>1 dollar	164	132 (80.5)	32 (19.5)	
**Total**	546	391 (77.3)	124 (22.7)	
**Characteristic**				
**Owns radio**				
Yes	308	232(75.3)	76(24.7)	.213
No	238	190(79.8)	48(20.2)	
**Owns mobile phone**				
Yes	234	191 (81.6)	43 (18.4)	.036
No	312	231 (74)	81 (26)	
**Adult female in the family**				
None	315	238 (75.6)	77 (24.4)	.408
>1	231	184 (79.7)	47 (20.3)	
**Covered by health insurance**				
Yes	170	126 (74.1)	44 (25.9)	.234
No	376	296 (78.7)	80 (21.3)	
**Nearest health facility**				
Health facility	452	344 (76.1)	108 (23.9)	.148
Dispensary	94	78 (83)	16 (17)	
**Total**	546	391 (77.3)	124 (22.7)	
**Characteristic**				
**Distance to nearest health facility, km**				
<1	259	188 (72.6)	71 (27.4)	.045
1-5	232	189 (81.5)	43 (18.5)	
>5	55	45 (81.8)	10 (18.2)	
**Attitude towards male involvement in childbirth**				
Negative	21	3 (14.3)	18 (85.7)	<.001
Positive	525	419 (79.8)	106 (20.2)	
**Subjective norms regarding male involvement in childbirth**				
Negative	21	4 (19)	17 (81)	<.001
Positive	525	418 (79.6)	107 (20.4)	
**Perceived behaviour control regarding male involvement in childbirth**				
Negative	17	4 (23.5)	13 (76.5)	
Positive	529	418 (79)	111 (21)	<.001

A large proportion (98.7%) of pregnant women had a positive attitude towards male involvement during childbirth. Most pregnant women also had positive perceived subjective norms (feeling male involvement is approved by relatives and friends) (93.4%), and positive perceived behaviour control (feeling able to engage in the behaviour) (98.7%) towards male involvement in childbirth. Similarly, many male partners had positive attitudes (96.2%), positive perceived subjective norms (96.2%) and positive perceived behaviour control (96.9%) towards male involvement in childbirth (Tables 1 and 2).

Among pregnant women, 71.6% stated that they intended to be accompanied by their male spouses during childbirth, while 77.3% of male partners stated that they intended to accompany their female partners during childbirth (Tables 1 and 2). In bivariate analysis, several factors were associated with women's intention to be accompanied by their male partner during childbirth, including, positive subjective norms (*P*=.006), ethnic group (*P*<.001), type of nearest health facility (*P*=.010) and parity (*P*=.014) (Table 1). Among male partners, positive attitude (*P*<.001), positive subjective norms (*P*<.001), positive perceived behaviour control (*P*<.001), ethnic group (*P*<.001), owning a mobile phone (*P*=.036) and walking distance to the nearest health facility (*P*=.045) all significantly influenced the intention to accompany their female partner during childbirth (Table 2).

In pregnant women, we were unable to develop a multivariable logistic model because of the very high proportion (>98%) of women represented in the category of ‘positive’ for attitudes and perceived behaviour control regarding male involvement in childbirth. We therefore calculated unadjusted ORs, which were: (OR 3.4, 95% CI, 0.8 to 15.5; *P*=.109) for positive attitude, (OR 0.2, 95% CI, 0.1 to 0.7; *P*=.012) for positive subjective norms and (OR 1.0, 95% CI, 0.2 to 5.3; *P*=.991) for positive perceived behaviour controls (data not shown). Thus pregnant women with positive subjective norms were 5 times less** likely to intend to be accompanied by their male partner during childbirth compared to women with negative subjective norms. Among male partners, a multivariable logistic model showed that positive attitudes (adjusted odds ratio [AOR] 9.0, 95% CI, 1.9 to 40.9; *P*=.004) and positive subjective norms (AOR 4.4, 95% CI, 1.1 to 18.6; *P*=.041) were significantly associated with increased odds of men's intention to accompany their partners during childbirth. Positive perceived behaviour control was not significantly associated with men's intention to accompany their partners during childbirth (data not shown).

## DISCUSSION

Our large, community-based study examined the influence of attitudes and beliefs on intentions for male involvement in childbirth in rural Tanzania, finding that over 90% of both female and male participants expressed positive attitudes, subjective norms and perceived behaviour control regarding male involvement in childbirth. However, these positive attitudes and beliefs did not always translate to intention; only about 70% of participants intending to be accompanied by their partner (or to accompany their female partner) during childbirth. Interestingly, the intention for male involvement in childbirth was greater among male partners (77%) compared to pregnant women (72%).

Our observation that female participants were less likely than their male partners to intend to have male involvement in childbirth suggests that, in the settings such as that of the study, women may be barriers to such involvement. This finding is contrary to another study conducted in a Maasai community in rural Tanzania, which reported that pregnant women are more in favour of being accompanied by their male partners compared to their male partners.^22^ This disparity in findings may be due to cultural differences.

In some rural communities, negative feelings towards male involvement may be rooted in local beliefs that pregnancy and childbirth are the sole responsibility of female partners.^12,13^

We conjecture that in this context, defiance against approval of others may have a stronger influence on the intention for male involvement in childbirth, compared to acceptance of approval of others. This idea is supported by our finding that among female study participants, positive subjective norms predicted a *lower* likelihood of intention for male involvement in childbirth, compared to negative subjective norms. This is contrary to the theory of planned behaviour, which postulates that societal approval of a behaviour promotes an individual's intention to perform the behaviour.^17^ Similarly, positive attitudes and perceived behaviour control regarding male involvement during childbirth did not significantly increase intention for male involvement among pregnant women, as would be predicted by the theory of planned behaviour. It is possible that other factors play a substantial role in predicting intention for male involvement during childbirth among pregnant women. Our findings suggest that interventions geared towards improving attitudes and beliefs about male involvement during childbirth may not be effective among pregnant women in some rural African settings.

In contrast to our findings among pregnant women, we found that among male partners, positive attitudes and positive subjective norms regarding male involvement significantly predicted intention to accompany female partners during childbirth. Male partners with a positive attitude were 9 times more likely to intend to accompany their female partners during childbirth compared to men with negative attitudes. Male partners who felt that others approved of their involvement during childbirth were 4 times more likely to intend to accompany their female partners during childbirth compared to men who felt others disapproved of their involvement. Our findings agree with those of other studies that report that male partners are in support of being involved in maternal services utilisation,^13^ and that beliefs and attitudes influence male intention to be involved in childbirth.

The study is not without limitations. Our study findings relied upon self-reported intention for behaviour, rather than actual behaviour. It is possible that respondents reported a behaviour that they believed would please the interviewer, rather than their true intentions regarding that behaviour. Previous studies suggest that intention for male involvement in childbirth may not match the actual male involvement in maternal services utilisation. Studies from rural Tanzania report that only 12% of male partners participate in maternal service utilisation^11^ and that cultural beliefs limit male involvement in childbirth to the provision of financial support for obstetric emergencies.^12^ It is unclear whether the low level of male involvement in childbirth is due to a lack of intent, or to other barriers, including financial constraints.^23^

## RECOMMENDATIONS AND CONCLUSION

Male partners were more likely than pregnant women to intend to have male involvement during childbirth. Among the 3 domains of intention in the theory of planned behaviour, only subjective norms influenced pregnant women's intention to be accompanied. Against expectation, positive subjective norms predicted a lower likelihood of intention among pregnant women. Among male partners, positive attitudes and positive subjective norms were statistically significant predictors of intention to accompany their female partners during childbirth. We recommend that interventional studies be conducted in rural African settings that target attitudes and subjective norms among men to increase male involvement during childbirth, and identify factors that positively influence women's intention regarding such involvement. Future studies in this area should assess objective behavioural endpoints.
